# Right heart failure syndrome

**DOI:** 10.18632/aging.101708

**Published:** 2018-12-13

**Authors:** Osama Soliman, Rahatullah Muslem, Kadir Caliskan

**Affiliations:** 1 Department of Cardiology the Thoraxcenter, Erasmus Medical center Rotterdam, The Netherlands; 2 Department of Cardiology, University Hospital of Antwerp, Belgium; 3 Department of Cardiothoracic Surgery, the Thoraxcenter, Erasmus Medical center Rotterdam, The Netherlands

**Keywords:** heart failure, right-sided heart failure, ventricular assist device, outcome

Left ventricular assist device (LVAD) implantation have become a standard therapy for selected patients with advanced heart failure (HF). Especially for older patients with end-stage HF who are deemed ineligible for heart transplantation, LVAD therapy has become their last resort. Though the survival of these patients on LVAD support is superior to optimal medical therapy, the use of LVAD is associated with high morbidity and mortality. Common complications include infection, bleeding, neurologic events, and right-sided heart failure (RHF). The latter has been shown in a cecent analysis of the largest European LVAD registry, the European registry of mechanical circulatory support (EUROMACS), to be prevalent in over 20% of patients in the early post-operative phase. RHF is furthermore, associated with an 18% excess in mortality at one year after LVAD implantation [1].

**Figure 1:**
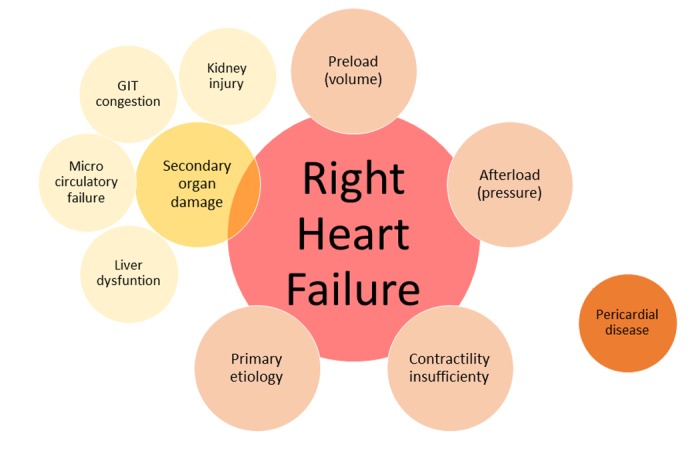
**The 5 pillars of right heart failure syndrome.** Intact pericardium or pericardial disease interact as an extra hemodynamic player of the ventricular interdependence. Abbreviation: GIT: gastrointestinal tract.

Several important predictors of RHF were identified during this study, including high right atrial to pulmonary capillary wedge pressure, low hemoglobin, need of multiple intravenous inotropes, INTERMACS Class 1-3, and severe RV dysfunction on echocardiography. These readily available pre-operative predictors were used to develop the EUROMACS-RHF risk score, a simple 5-item scoring system for the prediction of early RHF after LVAD implantation (Figure 1).

The RV is a non-spherical shaped thin muscle structure of which the function depends on multiple factors. In brief the RV performance depends on systemic venous return (pre-load), contractility of the RV free wall and interventricular septum, RV afterload, and pericardial compliance. Due to the inherent myofiber structure, the tri-chamber geometry of the RV, and the interdependent relationship of the RV and pulmonary circulation, the RV stroke volume is highly sensitive to changes in pressure (afterload) rather than volume. Thus, in contrast to a tolerant left ventricle, increase RV afterload could lead to significant drop in RV performance and thus reduced cardiac output.

The unique and complex interaction between the venous systemic and pulmonary circuits, the two components of the right heart circulatory system, makes the RHF syndrome a complex disease, which currently lacks a universal definition. This is reflected in the wide distribution of the incidence of RHF following LVAD implantation (4%-50%) [1]. A reasonable definition of severe RHF after LVAD, was used by our group for the EUROMACS-RHF risk score [1], as a combination of elevated central venous pressure and need for RV support; either mechanical via a RV assist device or pharmacological via the use of continuous intravenous inotropic support or pulmonary vasodilators such as inhaled NO.

The art of medicine is tackling RHF before it reaches the point of no return. The 5 common pillars of RHF therapy includes optimization of preload, contractility enhancement, afterload reduction, prevention of organ damage, and treatment of the underlying cause.

Improvement of the afterload can be achieved though reduction of pulmonary resistance via NO vasodilators. In addition, inotropic support or mechanical circulatory support can be utilized to enhance contractility. Late intervention is associated with secondary organ damage including venous and gastrointestinal congestions, acute kidney injury and liver dysfunction, perpetuating a vicious circle of further aggravating fluid retention and worsening RHF, eventually resulting in inevitable multi-organ failure. These grave outcomes are evident from the two-fold increased mortality after LVAD implantation in patients with RHF at six months. Emphasizing the need of pre-operative optimization of the RV function. However, previous risk scores for the prediction of RHF lacked discriminative power or external validation. The EUROMACS-RHF risk score, which outperformed previously published risk scores, could be utilized for the identification of candidates for RV support. Furthermore, the pre-operative risk determined with the EUROMACS-RHF risk score could assist in the decision process, preoperative preparation, and in determining the optimal timing of surgery. Finally, it is important to inform patients about their risk of RHF, especially in older patients receiving DT, considering there is no opportunity for bailout therapy for these patients.

The RV has been stated as the forgotten ventricle. Luckily, this young field in medicine enjoys arising interest and recognition of the devastating consequences of RHF. Building evidence and thus improving therapeutic strategies requires more research. Starting of with the identification of the natural history of RV changes after LVAD implantation. Furthermore, due to the fact that all therapeutic options for RHF remain empiric, clinical trials are warranted. Therefore, we recently set up a multicenter longitudinal study, the EuroEchoVAD Study (NCT03552679), aiming at clinical, hemodynamic, and echocardiographic quantification and prediction of the time course of RV function to ultimately redefine RHF in order to identify optimal management strategies after LVAD implantation. The enrollment in the EuroEchoVAD study is ongoing with an expected 600 patients at over 30 international sites.

In conclusion, the largest European registry of mechanical circulatory support revealed that RHF is very prevalent and manifest in the early period after LVAD implantation and that RHF was associated with an increased mortality. The EUROMACS-RHF risk score identifies high-risk patients for RHF in whom timely RV support should be considered. Clinical trials are warranted to determine optimal treatment strategies for patients at high-risk for RHF.
